# Aberrant seed development in *Litchi chinensis* is associated with the impaired expression of cell wall invertase genes

**DOI:** 10.1038/s41438-018-0042-1

**Published:** 2018-08-01

**Authors:** Jieqiong Zhang, Zichen Wu, Fuchu Hu, Lian Liu, Xuming Huang, Jietang Zhao, Huicong Wang

**Affiliations:** 1 State Key Laboratory for Conservation and Utilization of Subtropical Agro-bioresources, College of Horticulture, South China Agricultural University, Guangzhou, China; 2 Guangdong Litchi Engineering Research Center, College of Horticulture, South China Agricultural University, Guangzhou, China; 3 Key laboratory of tropical fruit tree biology of Hainan Province, Hainan Academy of Agricultural Science, Haikou, 571100 China; 4 Department of Life Sciences and Technology, Yangtze Normal University, Chongqing, China; 5 Present Address: Shanghai Center for Plant Stress Biology (PSC), Shanghai Institutes of Biological Sciences, Chinese Academy of Sciences, Shanghai, 201602 China

## Abstract

Cell wall invertase (CWIN) are known to play important roles in seed development. However, most reports to date have focused on a single gene family member, and have mainly investigated CWIN functions during the filling stage of seed development. In this study, we found significant lower levels of CWIN protein and activity associated with seed abortion in the *Litchi chinensis* cultivar “Nuomici.” We identified five litchi *CWIN* genes and observed that the expression of *LcCWIN5* was limited to the flower tissues and decreased sharply with fruit development. Silencing of *LcCWIN5* expression before 28 DAA (cell division stage) resulted in perturbed liquid endosperm development, smaller seeds, and higher seed abortion rate, while silencing after 28 DAA (filling stage) had no effect on seed development. In contrast, *LcCWIN2* was mostly expressed in the funicle and seed coat, and increased with fruit development. Decreased *LcCWIN2* expression and CWIN activity during early seed filling coincided with smaller seeds in the cultivar “Feizixiao.” Silencing of *LcCWIN2* caused a reduction in the seed size without inducing seed abortion. We propose that CWIN activity in seed maternal tissues during cell division stage is likely due to *LcCWIN5* expression, which regulates early seed development. On the other hand, CWIN activity during the filling stage is due to the expression of *LcCWIN2*, which may promote carbon import by creating a sucrose gradient. Comparable *LcCWIN5* expression, but much lower CWIN activity, detected in the funicle of “Nuomici” is consistent with post-translational regulation.

## Plant development: The complex control of seed size

A family of genes, known as cell wall invertases (*CWIN*s), control different stages of seed development in litchi, and could be used to breed more desirable fruits. Seed set and seed size are important characteristics of horticultural plants, with the seeds of some crops being significant food sources, while seedlessness or small seededness is sought after in many fruit crops. A team led by Jietang Zhao and Huicong Wang, of South China Agricultural University, studied the genes controlling seed set and size in small-seeded, large-seeded and seed-abortive cultivars of litchi (*Litchi chinensis*). They identified five members of the *CWIN* family, of which one, *LcCWIN5*, was shown to regulate early seed development, while another, *LcCWIN2*, was active during the later “seed-filling” stage. These results could aid the development of more attractive, small-seeded or seedless varieties of litchi.

## Introduction

Angiosperm seeds are important sources of food and animal feed. They are composed of the two fertilization products, the embryo and the endosperm, surrounded by the maternally derived seed coat. Coordinated interaction between all three seed components is a requirement for seeds to complete their development^1^.

Molecular genetic studies have shown that cell wall invertase (CWIN) proteins play a key role in determining the sequential development of the endosperm and the embryo^2^, and a positive correlation has been observed between CWIN activity and seed development in a range of plant species including bean (*Vicia faba*)^3,4^, barley (*Hordeum vulgare*)^5^, maize (*Zea mays*)^6^, rice (*Oryza sativa*)^7^, tomato (*Solanum lycopersicum*)^8^, cotton (*Gossypium hirsutum*)^9^, and soybean (*Glycine max*)^10^. Mutants of maize and rice with reduced *CWIN* expression produce smaller seeds^7,11^, whereas elevation of CWIN activity by silencing its inhibitor in tomato increases the seed size^12^.

CWIN is central to phloem unloading and carbon partitioning, especially in cellular sites lacking symplastic connections such as the filial tissues of seeds^5^ and plays a critical role in photoassimilate import in the developing seeds of maize^11^, barley^5^, bean, and tomato^4,13^. High CWIN activities correlate with high glucose levels, generally favoring cell division and promoting cell differentiation during seed development^14^. Thus, CWIN has been associated with seed development by regulating carbon import and/or cell division of filial tissues through sugar signaling.

CWIN multigene families of varying sizes in a number of plants have been described^15–18^. However, most such studies to date have focused on a single CWIN gene family member, and have investigated its role mainly in the filling stage of seed development. In contrast, there are fewer studies on CWIN during early seed development, when the endosperm enters a phase of substantial nuclear division immediately after fertilization, along with rapid growth of the seed integuments. Early seed development, or seed set, is a critical stage that determines the yield potential. At this stage, seed development is highly sensitive to biotic or abiotic stresses, which can lead to abnormal development or even abortion, and consequently irreversible yield loss^9^. In this regard, a growing body of literature suggests that invertases are a central component of the plant response to biotic and abiotic stimuli^19^. Determining how *CWIN* genes regulate seed development will not only advance the understanding of plant reproductive biology, but may also suggest strategies to manipulate crop yield and improve both fruit quality and tolerance to stress.

Litchi (*Litchi chinensis* Sonn.) is an evergreen fruit crop that is widely cultivated in warm regions of the world. The production of small sterile seeds is an economically desirable trait of litchi fruits, and cultivars have been bred with small seeds or stenospermy, resulting in fruits with greater commercial value. In a previous study, we demonstrated that seed abortion in a stenospermic litchi cultivar, “Nuomici,” might be due to low CWIN activity in seed pedicel and seed coat^20^. Larger seed, longer developmental period, and easily separated seed pedicel (funicle) make litchi an excellent model for exploring the roles of CWIN in seed development. In addition, an investigation into the stenospermy mechanism in litchi has the potential to expand our knowledge of the formation of stenospermic fruit and to provide an important reference for genetic improvement programs, and help to manage fruit set.

In this current study, big-seeded, small-seeded, and seed-aborted litchi cultivars were used to investigate the role of different *CWIN* genes in seed development. To this end, we measured CWIN activities in the funicle, seed coat, and cotyledon of different cultivars and detected the presence of CWIN proteins using immunocolloidal gold labeling in the funicle. Five litchi *CWIN* genes (*LcCWIN1-5*) were identified and characterized, and the roles of two of them in seed development were evaluated by suppressing their expression through virus-induced gene silencing (VIGS). We sought to answer the following questions: (i) does CWIN regulate seed development of litchi? (ii) which *CWIN* gene family member is critical for the normal development of litchi seeds? (iii) do *CWIN* genes affect the early or late stage of seed development?

## Results

### A distinct developmental transition between the cell division and filling stages, and the presence of less liquid endosperm in stenospermic “Nuomici”

According to Lü et al.^21^, double fertilization takes place in litchi 2–3 days after pollination, followed by division of the nuclei of the primary endosperm, which initially develops as a syncytium, leading to the formation of a large cell containing multiple nuclei surrounding the central vacuole (liquid endosperm). The zygote begins to divide after 3–7 days and reaches the heart stage around 30 days after anthesis, when the liquid endosperm is substantial^21^.

**Fig. 1:**
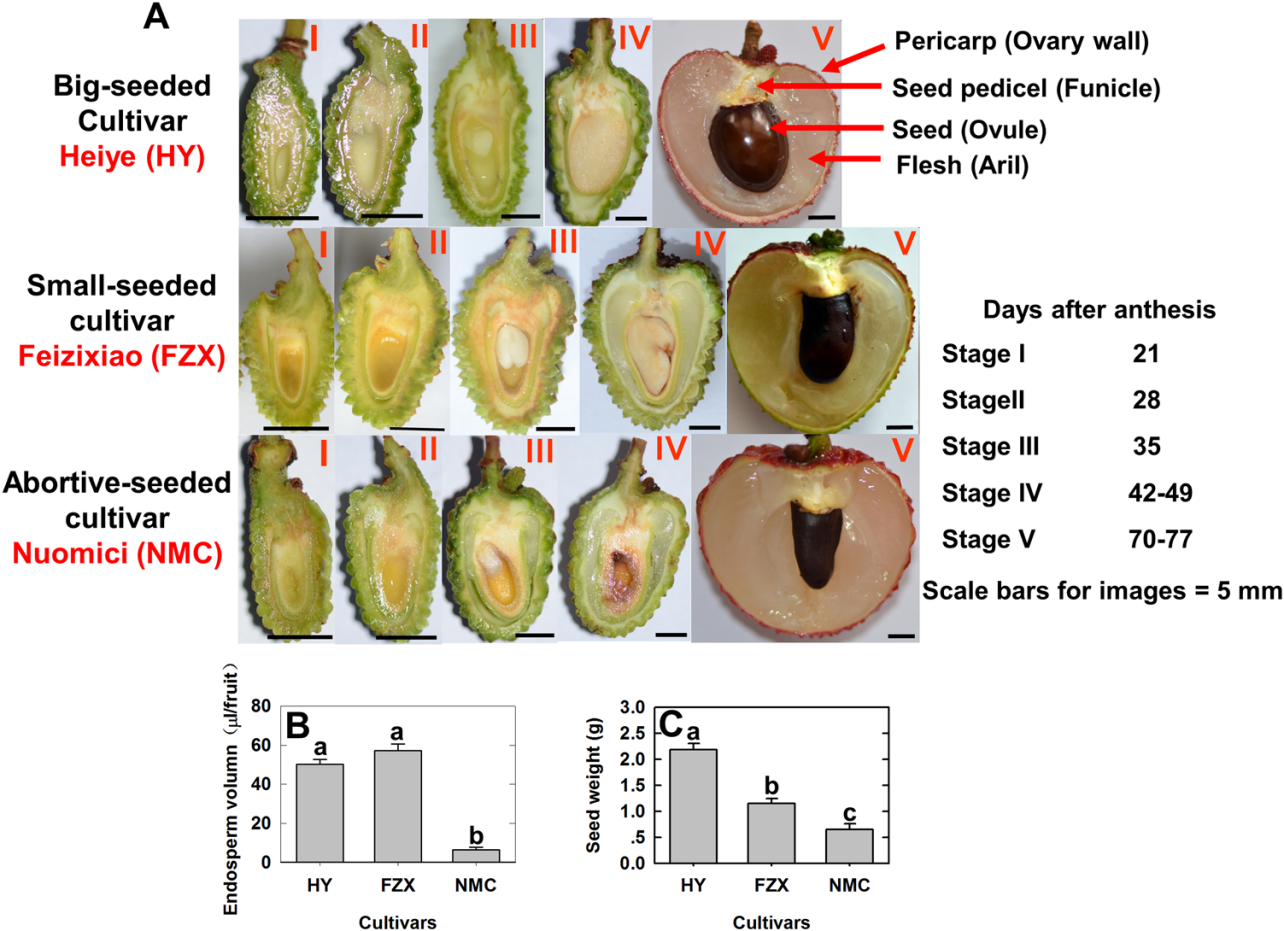
Liquid endosperm, embryo, and cotyledon development in the litchi cultivars “Heiye” (HY), “Feizixiao” (FZX), and “Nuomici” (NMC). **a** Images of longitudinal sections of seeds. Stage I, 21 days after anthesis (DAA); Stage II, 28 DAA; Stage III, 35 DAA; Stage IV, 42 DAA for HY, 49 DAA for FZX and NMC; Stage V, 70 DAA for cultivars HY and FZX, 77 DAA for NMC. **b** Liquid endosperm volume inside the seeds at 28 DAA. **c** Seed weight at fruit maturity. Different letters above the bars represent significant differences at *P* < 0.05 (*n* = 3, Duncan’s multiple range test)

As shown in Fig. 1a, in both, a big-seeded cultivar, “Heiye” (HY), and a small-seeded cultivar, “Feizixiao” (FZX), visible liquid endosperm was observed around 21 days after anthesis (DAA, Stage I) and the volume increased rapidly over the next week. The liquid endosperm was most abundant at 28 DAA (Stage II) when the embryo reached the heart stage with a rudimentary cotyledon, and after 28 DAA, the liquid endosperm was absorbed by the quickly developing cotyledon. Seeds with a much larger embryo and cotyledons, but less liquid endosperm were observed at 35 DAA (Stage III). Thus, 28 DAA represents a transition point between the cell division stage and the filling stage during litchi seed development. In the seed-aborting cultivar, “Nuomici” (NMC), sequential liquid endosperm and embryo development was not observed (Fig. 1a), and the amount of liquid endosperm at 28 DAA was much lower in NMC than in HY or FZX (Fig. 1b). The three tested cultivars displayed significant differences in seed size at maturity (Fig. 1c). HY produced larger seeds with normally developed embryos and cotyledons, and FZX had smaller seeds with normally developed embryos, but smaller cotyledons, while NMC produced seeds without embryos or cotyledons.

### Differences in the levels of CWIN protein and activity

**Fig. 2:**
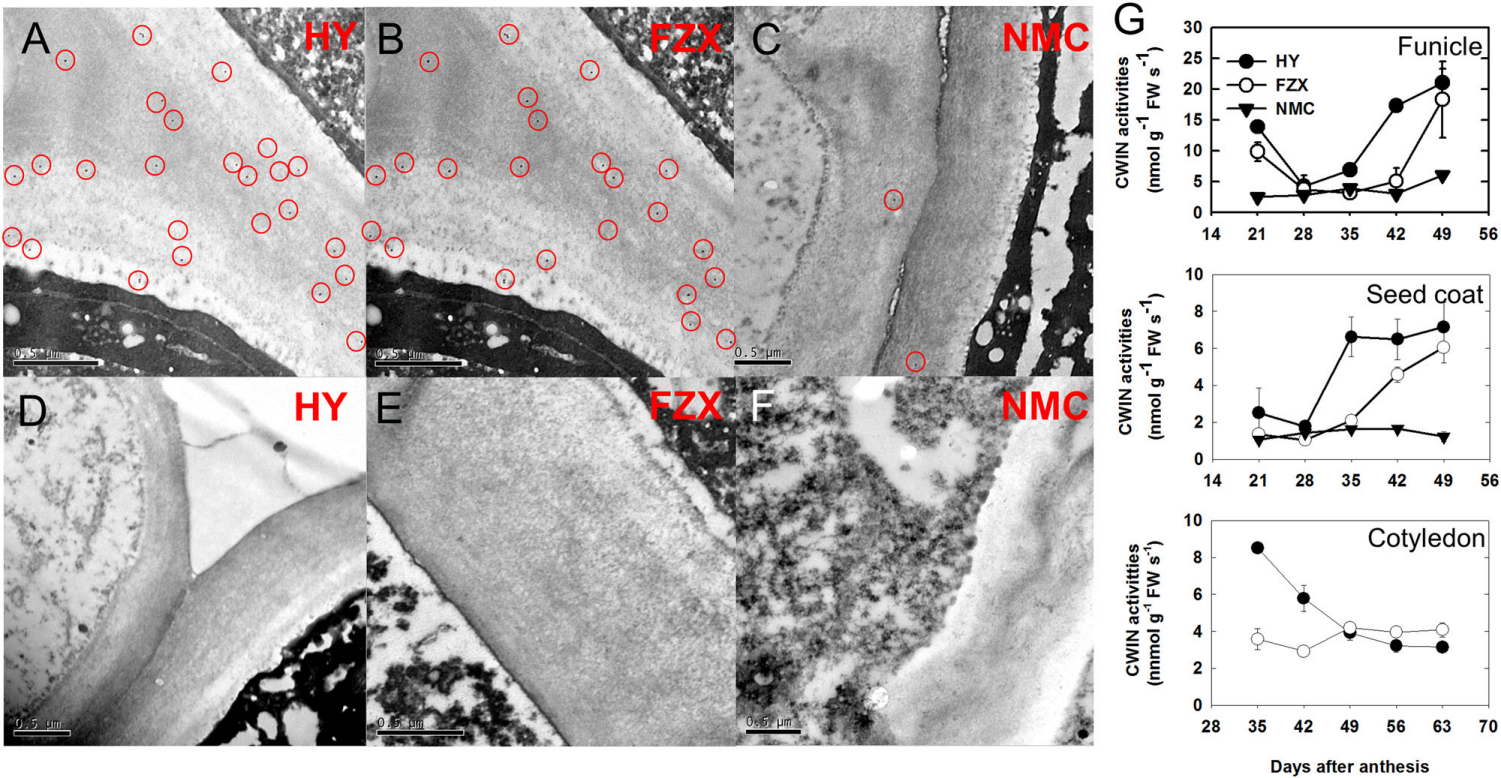
Immunogold localization and density of the putative CWIN proteins and CWIN activities in the funicle, seed coat, and cotyledon of litchi. **a**–**c** The CWIN antiserum labeling in the cell wall of the three tested cultivars. **d**–**f** Control cell walls of the three cultivars tested. **g** CWIN activities in the extracts from the funicle, seed coat, and cotyledon during critical seed developmental stages. The error bars represent the standard error of the three replicates

We used a CWIN antiserum coupled with immunogold labeling to investigate the presence CWIN proteins in the funicle at the early stage of fruit development (7 DAA). In contrast to the scarcely found immungold particles in a control without antiserum, the proteins reacting with anti-CWIN serum were clearly observed in the cell walls of all the three cultivars tested (Fig. 2a–f). The density of immunogold particles was significant lower in the cell walls of NMC than in those of FZX and HY. In addition, we measured the CWIN activities in protein extracts from the funicle, seed coat, and cotyledon, and detected significantly lower activity in NMC than in HY and FZX at most sampled points (Fig. 2g). These results were consistent with the lower fructose and glucose contents observed in the funicles of NMC, compared to those of FZX and HY^22^. In HY and FZX, the funcile CWIN activities were high at 21 DAA, followed by a short decrease and an increase again at 28 DAA and 42 DAA, respectively. From 35 to 42 DAA, the CWIN activities in the funicle, seed coat, and cotyledon of FZX were much lower than those in HY (Fig. 2g).

### Identification and characterization of the Litchi *CWIN* gene family

In order to identify litchi *CWIN* genes, sequences annotated as putative glycoside hydrolases were obtained by searching the *L*. *chinensis* genome database (http://litchidb.genomics.cn/page/species/index.jsp) and CWIN-specific regions including conserved functional motifs and a signal peptide were analyzed. Five putative *CWIN* genes, named *LcCWIN1-5,* were identified, and full-length cDNAs of each were cloned by RT-PCR using gene-specific primers. The cDNA and deduced amino acid sequences were deposited in the GenBank with the following accession numbers: LcCWIN1 (KX981206), LcCWIN2 (KX981207), LcCWIN3 (KX981208), LcCWIN4 (KX981209), and LcCWIN5 (KX9812010).

**Fig. 3:**
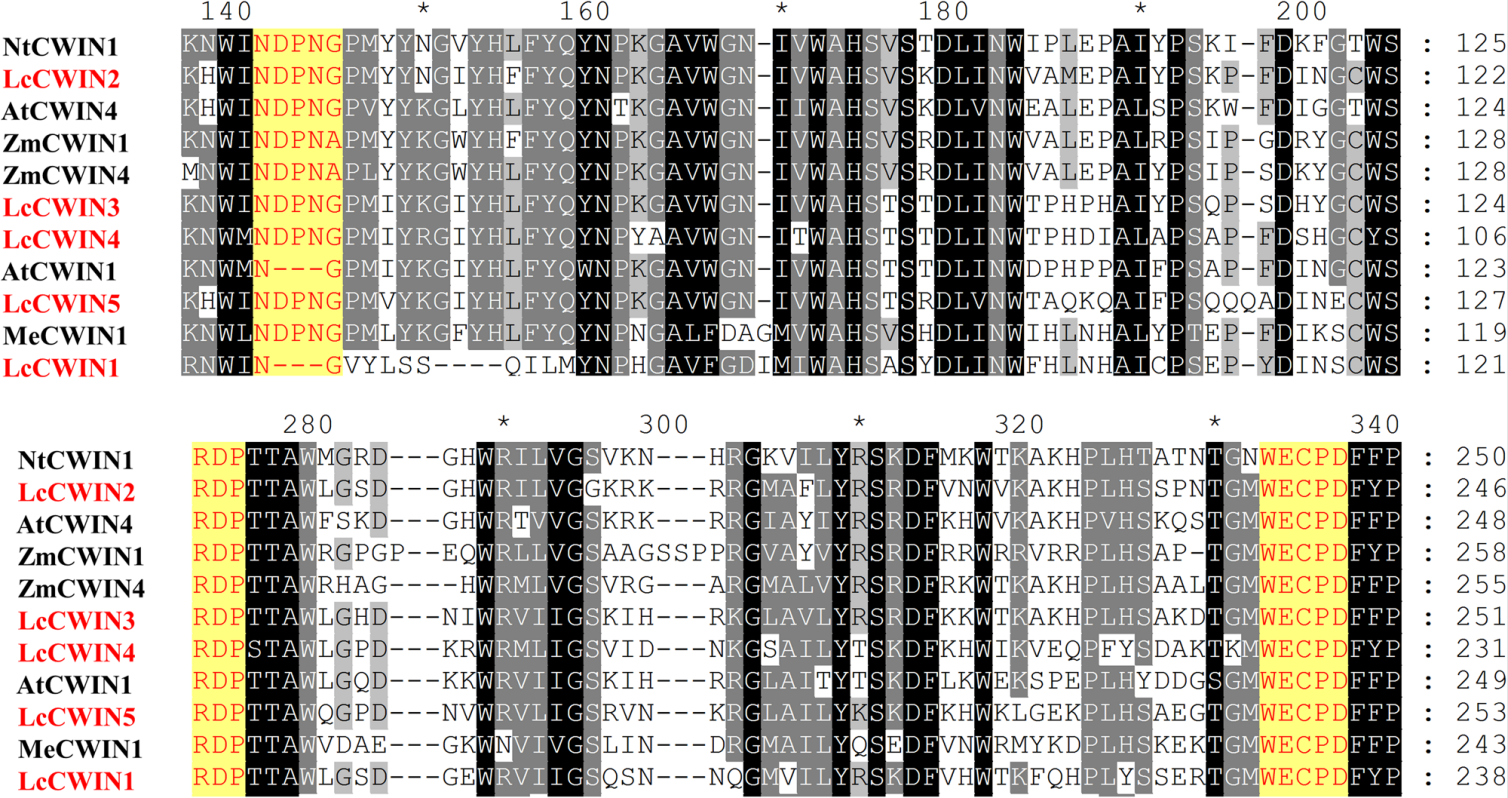
Protein alignment and domain structure of the LcCWIN proteins with other plant CWIN proteins (in GenBank). *Arabidopsis thaliana* (AtCWIN1, NP_001189881; AtCWIN4, NP_565837), *Manihot esculenta* (MeCWIN1, AFH77950), *Nicotiana tabacum* (NtCWIN1, X81834), and *Zea mays* (ZmCWIN1, AF050631; ZmCWIN4, AF043347). Three conserved sequence domains: NDPNG (β-fructosidase motif), RDP, and WECP(V)D, which correspond to the predicted active sites of the enzyme, are shaded in yellow with red letters

**Fig. 4:**
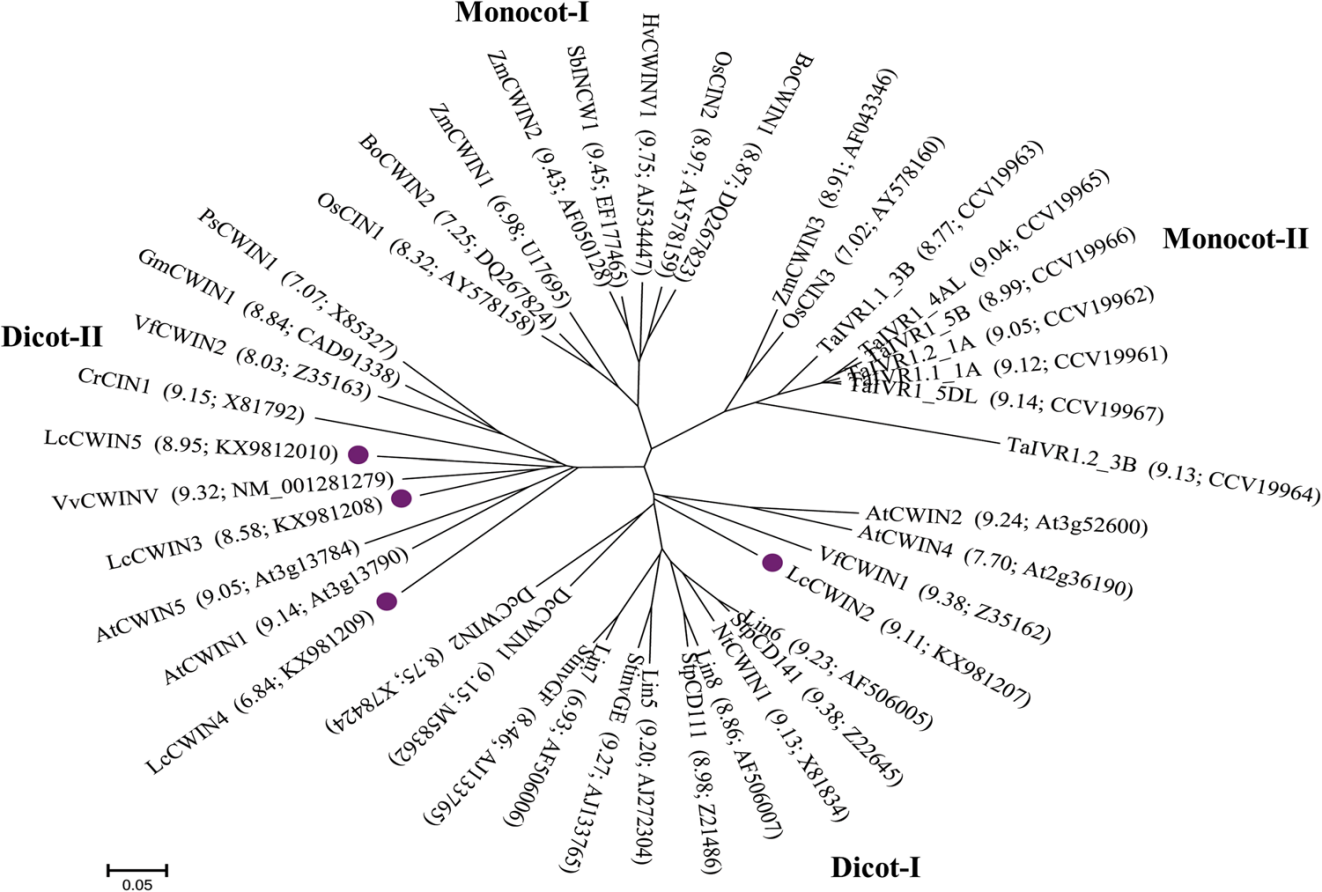
Phylogenetic tree of the CWIN proteins constructed using the neighbor-joining method. The scale bar corresponds to a distance of five changes per 100 amino acid positions. For each protein, the pI value and accession number are shown in parentheses. Species abbreviations: At *Arabidopsis thaliana,* St *Solanum tuberosum*, Nt *Nicotiana tabacum*, Vf *Vicia faba*, Dc *Daucus carota*, Zm *Zea mays*, Os *Oryza sativa,* Hv *Hordeum vulgare,* Sb *Sorghum bicolor,* Gm *Glycine max,* Cr *Chenopodium rubrum*, Ps *Pisum safivum*, Bo *Bambusa oldhamii,* Gh *Gossypium hirsutum*, Ta *Triticum aestivum*, and Vv *Vitis vinifera*

The predicted open reading frames (ORFs) of the five genes were between 1677 and 1740 bp, corresponding to deduced amino acid sequences ranging from 558 to 579 (Table 1). The most homologous sequences in the GenBank, identified using BLAST (https://blast.ncbi.nlm.nih.gov), ranged from 69 to 79% identity (Table 1). The sequences of *LcCWIN2* and *LcCWIN5* ORFs were identical, while other *LcCWINs* displayed 2–7 SNPs among the three cultivars tested. With the exception of a three amino acid deletion in the NDPNG motif (β-fructosidase motif) in LcCWIN1, the three conserved sequence domains that represent the predicted active sites (i.e., NDPNG, RDP, and WECP[V]D) of the five LcCWIN proteins were intact (Fig. 3). To investigate the phylogenetic relationship of the LcCWIN proteins with homologs from different plant species, an unrooted phylogenetic tree was constructed using the neighbor-joining method (Fig. S1). The sequences were classified into five major groups: monocot-I, monocot-II, dicot-I, dicot-II, and CWINs with an acidic pI from both dicots and monocots. LcCWIN1 belonged to the subgroup with an acidic pI, together with *Arabidopsis* *thaliana* AtCWIN6, and maize ZmCWIN4. CWIN proteins characterized by a high pI are thought to interact with the cell wall, while invertases with an acidic pI are thought to be soluble and localized intracellularly^12,23,24^. Another phylogenetic tree was constructed without CWIN proteins with acidic pI (Fig. 4), and LcCWIN2 was found to belong to the dicot-I group, along with *A*. *thaliana* AtCWIN2/4, as well as sequences from tomato (Lin5-8) and potato (StinvGE, StinvGF, StpCD111 and StpCD141). LcCWIN3-5 and *A*. *thaliana* AtCWIN1/5 grouped with other dicot-II proteins, with LcCWIN3/5 being most closely related to the CWIN from grapevine.

**Table 1:** Sequence homologies based on amino acid sequences of litchi CWIN isolated from litchi cv. “Feizixiao,” and single-nucleotide polymorphism (SNP) numbers detected in LcCWINs, among the three cultivars tested

Gene	GenBank number	ORF (bp)	Length (AA)	Top BLAST match (GenBank number)	Homology (%)	SNP numbers
*LcCWIN1*	KX981206	1731	576	*Theobroma cacao* (XM_018128260.1)	69	2
*LcCWIN2*	KX981207	1722	573	*Citrus sinensis* (XM_006481682.1)	75	0
*LcCWIN3*	KX981208	1734	577	*Citrus sinensis* (XM_006426486.1)	79	7
*LcCWIN4*	KX981209	1677	558	*Dimocarpus longan (*KP769771.1)	72	2
*LcCWIN5*	KX981210	1740	579	*Theobroma cacao* (XM_007024428.2)	69	0

### *LcCWIN* expression patterns and CWIN activities

**Fig. 5:**
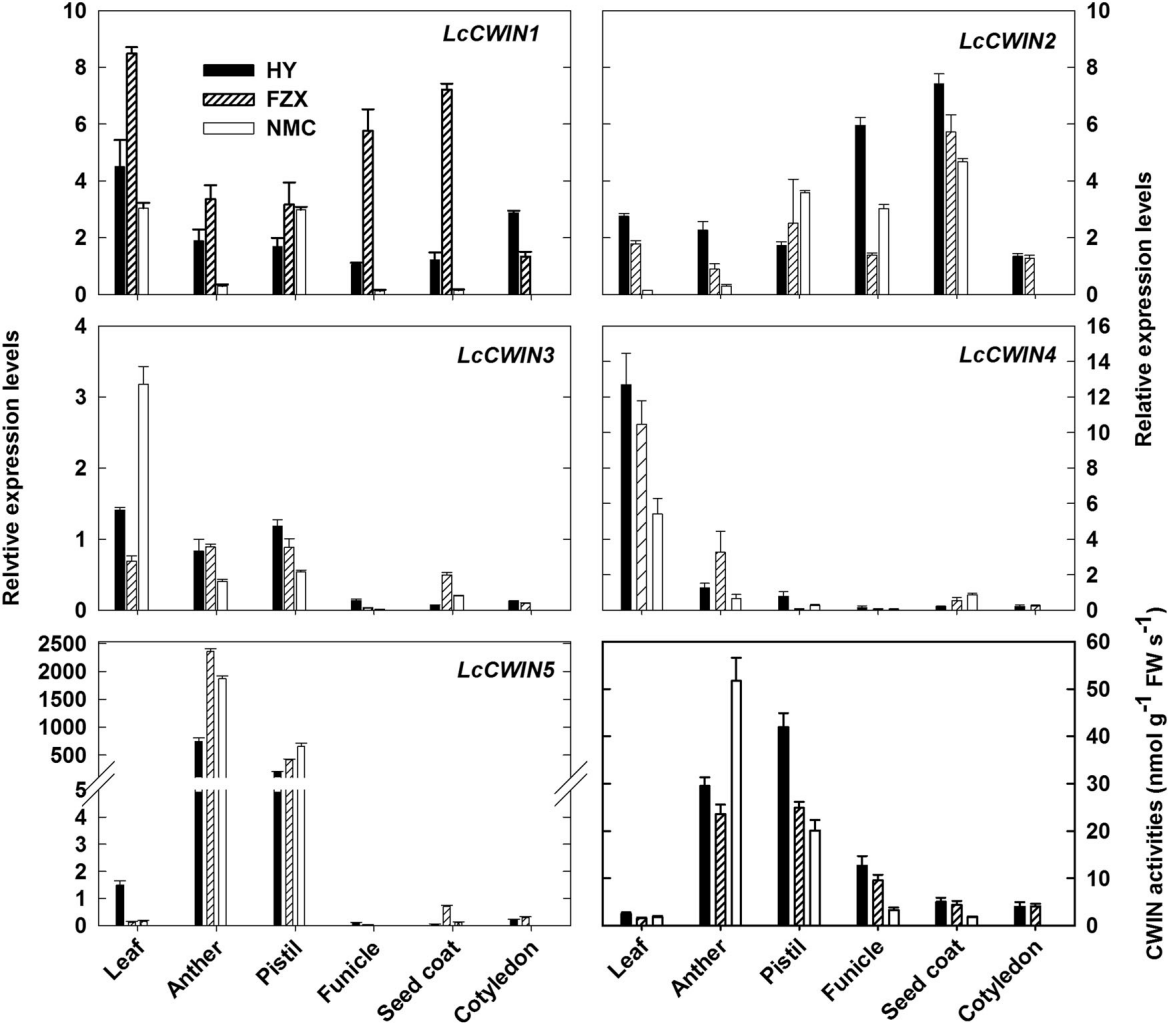
The expression patterns of the five litchi *CWIN* genes and CWIN activities in different organs/tissues. New mature leaves were taken from the first autumn shoots. Anther and pistil were taken on the day of full bloom, while funicle, seed coat, and cotyledon were sampled from fruits at 49 days after anthesis. The error bars represent the standard error of three replicates

The transcript levels of the *LcCWIN* genes and the CWIN activities in different litchi tissues were measured (Fig. 5), and we observed notable differences. *LcCWIN1*-*4* were expressed in all tissues, while *LcCWIN5* was specifically expressed in anthers and pistils. Among the four constitutively expressed *LcCWIN* genes, we observed that *LcCWIN2* was predominantly expressed in the funicle and the seed coat, and so we focused and targeted this gene in subsequent studies of the role of CWIN genes in seed development. *LcCWIN1* also showed relatively high expression levels in the funicle and seed coat, but the predicted protein had a three amino acid deletion in the β-fructosidase motif. The expression pattern of *LcCWIN5* was generally consistent with that of LIN5, a tomato CWIN gene that was shown to be required for seed development^8^. Thus, *LcCWIN2* and *LcCWIN5* were selected for further studies. Consistent with the predominant *LcCWIN5* expressions, anther and pistil displayed the highest CWIN activities, followed by the funicle and the seed coat (Fig. 5).

**Fig. 6:**
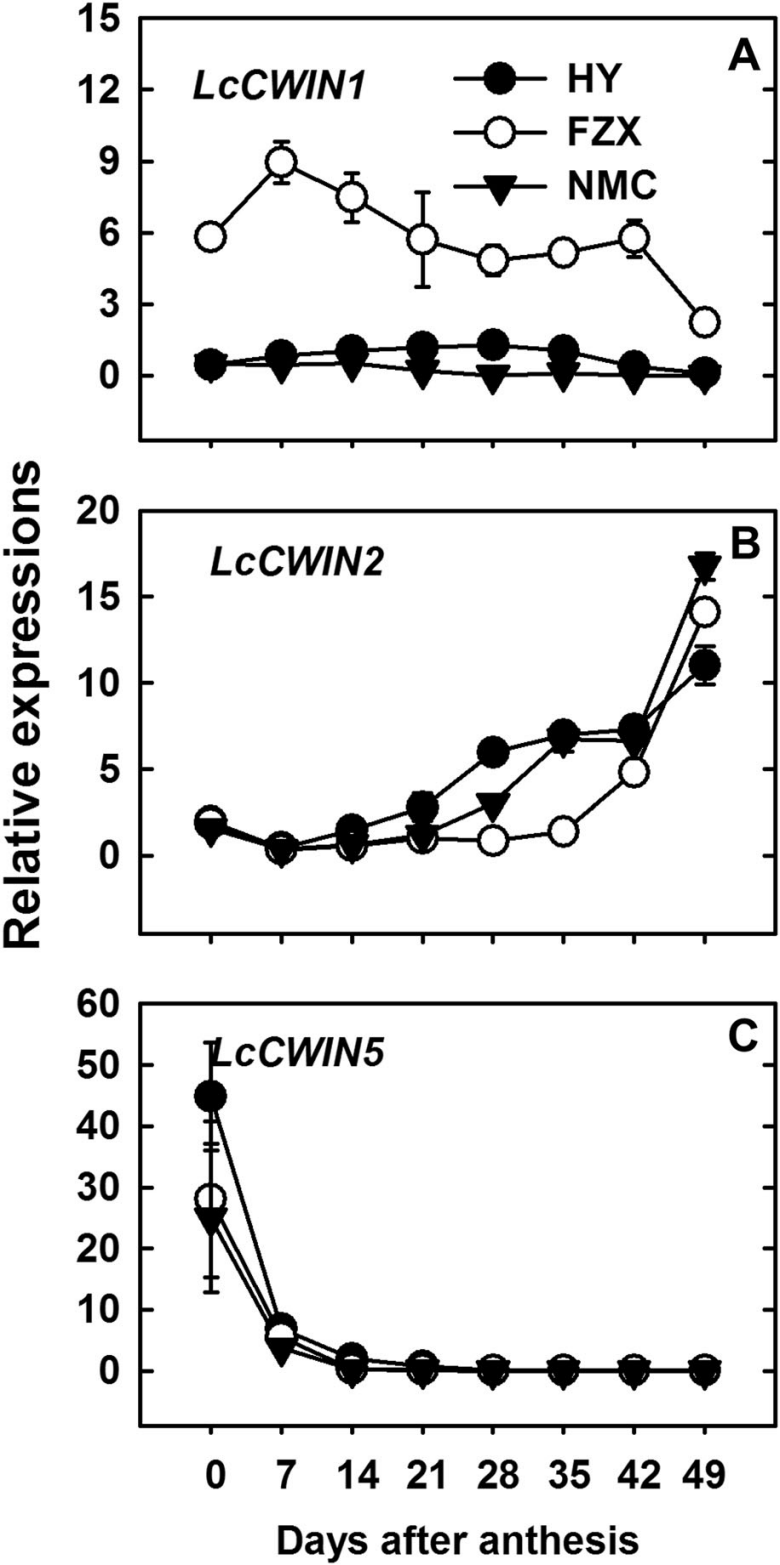
Changes in the expression of *LcCWIN1* (**a**), *LcCWIN2* (**b**), and *LcCWIN5* (**c**), as determined by quantitative real-time PCR in the funicle of three litchi cultivars during fruit development. The *LcActin* (HQ615689) and *LcGAPDH* (JF759907) genes were used to normalize gene expression under identical conditions. The error bars represent the standard error of three replicates

The developmental patterns of *LcCWIN1*, *LcCWIN2*, and *LcCWIN5* expression in the funicle of the cultivars tested are shown in Fig. 6a–c. *LcCWIN1* was preferentially expressed in FZX throughout fruit development. The expression of Lc*CWIN2* remained low before 14 DAA in all the three cultivars tested. Increased expression occurred later, but at different time points in different cultivars. A noticeable increase in *LcCWIN2* expression was evident at 21 DAA in HY, at 28 DAA in NMC, and at 42 DAA in FZX. In the funicle of all the cultivars, the expression of *LcCWIN2* increased, while that of *LcCWIN5* decreased with seed development. *LcCWIN5* expression peaked at the day of blooming, followed by a sharp decrease with seed development.

### In vivo functional analysis of *LcCWIN2* and *LcCWIN5* using VIGS in normal seed cultivars

VIGS is a powerful and rapid tool that enables targeted gene loss-of-function studies, and has been successfully applied to induce the silencing of phytoene desaturase (PDS, EC 1.3.99.30) gene in woody litchi leaves and of *LcUFGT1* in the pericarp^25^. In the present study, to test the function of *LcCWIN2* and *LcCWIN5* in litchi seed development, the vectors pTRV2-LcCWIN2 and pTRV2-LcCWIN5 were generated by inserting fragments of *LcCWIN2* or *LcCWIN5* into the pTRV2 vector. An *Agrobacterium tumefaciens* suspension containing the pTRV2-LcCWIN2 or pTRV2-LcCWIN5 constructs was injected into the fruit stalks still attached to the plant at 21–35 DAA, or wetted the panicles on the day of female blooming.

**Fig. 7:**
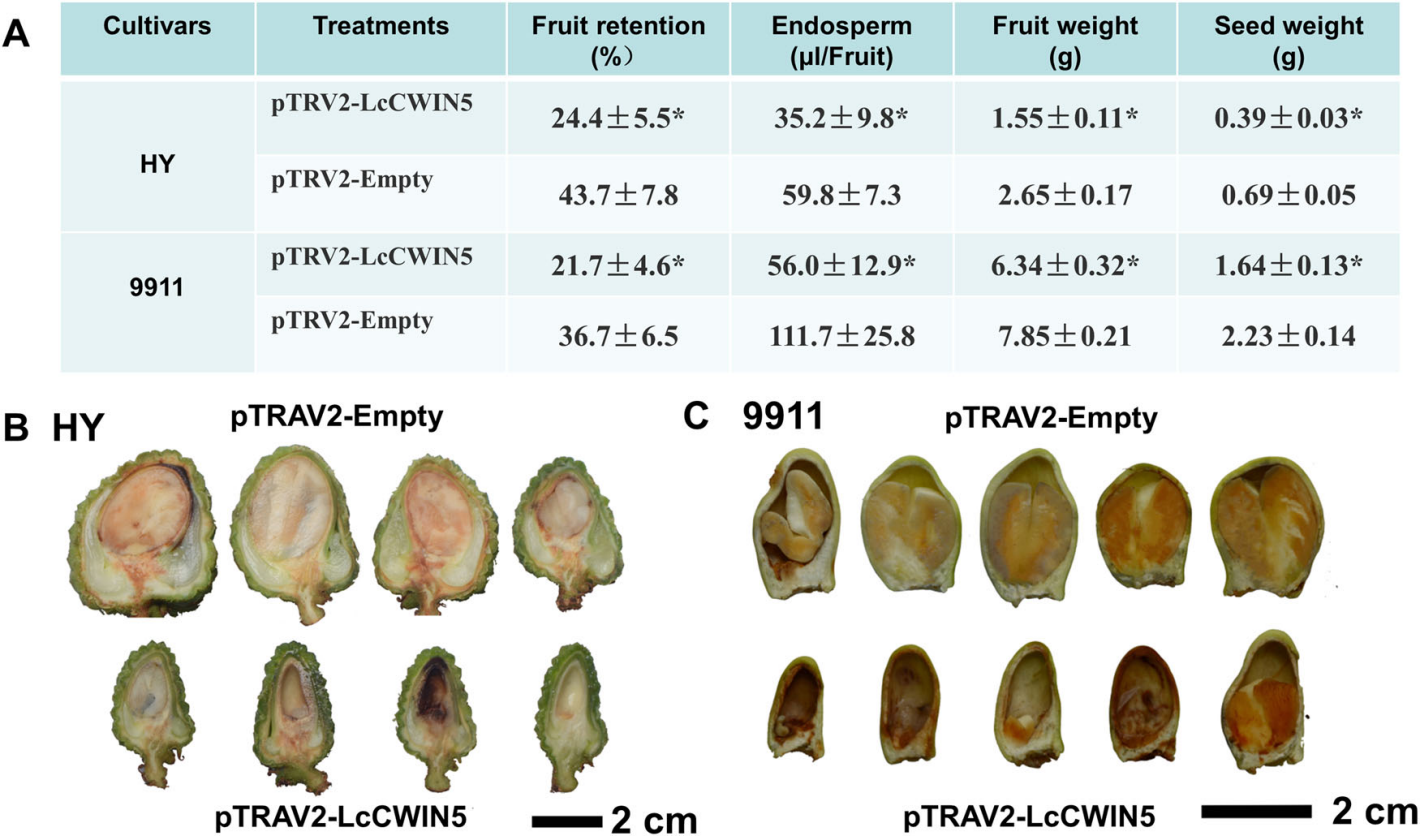
Effect of VIGS-mediated *LcCWIN5* gene silencing on seed development of big-seeded litchi cultivars, HY, and 9911. **a** The effects of *LcCWIN5* silencing through fruit-stalk injection at 21 days after anthesis on the fruit retention rate, liquid endosperm volume, and fruit and seed development. Liquid endosperm volume was investigated at 10 days after the treatments. Fruit retention, fruit and seed development were investigated at 3 weeks after the treatments for HY, but at 5 weeks after the treatments for 9911. *represent significance at *p* < 0.05 using *T*-test (*n* = 20)

First, we explored the effects of fruit-stalk injection with pTRV2-LcCWIN5 at 21 DAA on seed development of normal seed litchi cultivars, HY and 9911 (Fig. 7). Ten days after the treatment, the pTRV2-LcCWIN5 infiltration resulted in a significantly lower fruit retention rate and liquid endosperm volume (Fig. 7a). At 3 weeks (cultivar HY) or 5 weeks (cultivar 9911) after the injection, significantly lower fruit and seed weight were observed in fruit that had been infiltrated with pTRV2-LcCWIN5. Longitudinal sections confirmed a higher incidence of embryo and cotyledon development failure in fruits treated with pTRV2-LcCWIN5 than those treated with pTRV2-Empty control vector (Fig. 7b, c).

We also silenced the expression of *LcCWIN2* and *LcCWIN5* through panicle dipping and examined the effects on seed development in the cultivars “Heiye” (HY) and “Baitangying.” To confirm the suppression effects, we performed quantitative RT-PCR and measured CWIN enzyme activities in different fruit tissues of HY at 4 weeks after panicle dipping (Fig. S2). The primers that annealed to a region outside the *LcCWIN2* and *LcCWIN5* sequences were used for silencing. We observed that in the pTRV2-LcCWIN5-infiltrated fruits, the *LcCWIN5* transcript levels were significantly reduced in the funicle, when compared with the pTRV2-Empty control (Fig. S2A). The ineffective silencing in the other tissues might reflect the extremely low expression of the *LcCWIN5* gene in those tissues. In contrast, *LcCWIN2* expression was reduced in the funicle, seed coat, and cotyledon of the pTRV2-LcCWIN2-infiltrated fruit, compared with the fruit infiltrated with the control vector (Fig. S2B). Both the silencing of *LcCWIN5* and *LcCWIN2* resulted in decreased CWIN activities in the funicle, seed coat, and cotyledon, although the decrease was considerably greater in the case of *LcCWIN5* silencing (Fig. S2C–E). In both cultivars, the reduction in *LcCWIN2* transcript levels did not affect the fruit retention and seed abortion rate, but significantly reduced the seed weight, while the silencing of *LcCWIN5* led to significantly lower fruit retention, liquid endosperm volume, and seed weight, but a higher seed abortion rate (Table 2).

**Table 2:** The effects of *LcCWIN2* and *LcCWIN5* silencing, through panicle dipping in a bacterial suspension at female blooming, on the fruit retention rate and seed development of the litchi cultivars “Heiye” and “Baitangying”

Cultivar	Treatments	Fruit retention (%)	Liquid endosperm (μl/seed)	Seed weight (g)	Abortion rate (%)
Heiye	pTRV2-LcCWIN2	23.7 ± 4.5b	50.2 ± 2.5a	1.37 ± 0.11b	19.2 ± 5.1b
	pTRV2-LcCWIN5	7.5 ± 1.4a	38.6 ± 5.5b	0.73 ± 0.10c	50.3 ± 11.2a
	pTRV2-Empty	25.6 ± 6.6b	54.6 ± 5.3a	1.87 ± 0.14a	12.4 ± 3.5b
Baitangying	pTRV2-LcCWIN2	38.2 ± 3.8a	13.3 ± 0.76a	0.75 ± 0.02b	51.3 ± 2.3b
	pTRV2-LcCWIN5	33.7 ± 4.2a	9.0 ± 0.61b	0.70 ± 0.04b	69.4 ± 4.8a
	pTRV2-Empty	38.8 ± 4.4a	15.2 ± 0.68a	0.86 ± 0.08a	50.0 ± 5.0b

Liquid endosperm volume was investigated at ten days after treatments. Fruit retention was investigated at three weeks after treatments. Seed weight and abortion rate were investigated at maturity. Different letters after the values in the same cultivars indicate significant differences at *P* < 0.05 among treatments, according to Duncan’s Multiple New Range Test (*n* = 20).

**Fig. 8:**
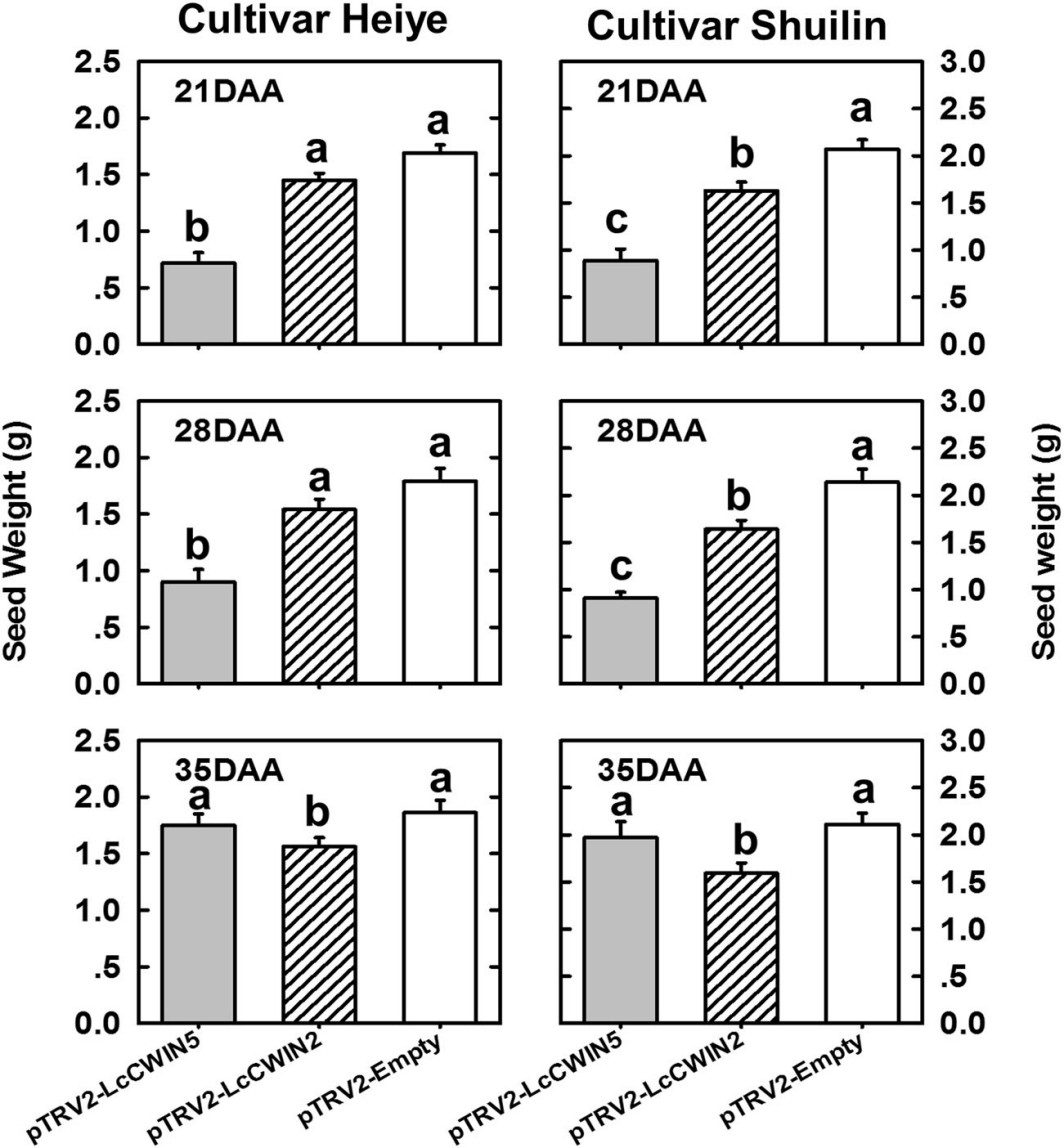
Effects of VIGS-mediated *LcCWIN5* and *LcCWIN2* gene silencing at three different fruit developmental stages on the seed size of the big-seeded litchi cultivars, “Heiye” and “Shuilin.” Injection was given at 21, 28, and 35 days after anthesis, and the seeds were harvested at fruit maturity (77 days after anthesis). Different letters above the bars represent significant differences at *P* < 0.05 (*n* = 20, Duncan’s multiple range test)

When *LcCWIN2* and *LcCWIN5* were silenced through fruit-stalk injection at different stages of seed development in two big-seeded cultivars, “Heiye” and “Shuilin,” we observed different effects on the seed weight depending on the silenced gene and the developmental stage (Fig. 8). The fruits infiltrated with a *LcCWIN5* antisense construct at 21 and 28 DAA produced significantly smaller seeds, while the silencing at 35 DAA did not significantly affect the seed weights in both cultivars. On the other hand, the silencing of *LcCWIN2* at 21, 28, or 35 DAA decreased the seed weight in both cultivars, although it was less apparent as comparing to the silencing of *LcCWIN5*.

## Discussion

### CWIN regulates early seed development

Seed development depends on the coordinated development of the seed coat, endosperm, and embryo. In the stenospermic litchi cultivar NMC, a normal seed coat develops, but visible liquid endosperm development does not occur and zygote development is retarded (Fig. 1). In parallel with the failure of sequential liquid endosperm and embryo development, we saw a significant lower density of CWIN-immunogold particles and reduced CWIN activities at the site of seed assimilate unloading, compared with the big-seeded cultivar, HY, and the small-seeded cultivar, FZX (Fig. 2). Furthermore, silencing of *LcCWIN*5 resulted in decreased CWIN activity, a significantly lower liquid endosperm volume, and a higher seed abortion rate in the big-seeded cultivars (Table 2 and Fig. 7). *LcCWIN5* expression in the funicle, but not in the cotyledon, was significantly reduced, paralleling with the perturbed seed development in response to *LcCWIN5* silencing (Fig. S2). These results suggest a key role for maternal CWIN in litchi seed development.

There is considerable evidence of CWIN playing a role in seed development^3–10^. However, these studies mainly focused on the filling stage, and the underlying mechanism of CWIN in regulating seed development was unclear. In the present study, litchi seed development could be clearly divided into the cell division stage and the filling stage at around 28 DAA, when the zygote reaches the heart stage (Fig. 1). There is compelling evidence that CWIN activity correlates with mitotic activity, cell number, and seed size. In large-seeded genotypes of fava bean (*Vicia faba*), CWIN is active for longer time in the enlarged seed coat and, consequently, more cells are produced in the embryo^5^. Additionally, in maize, a CWIN deficiency mutation (*mn1*) impairs endosperm development by reducing the cell number^6,26^. It is also noteworthy that the effect of CWIN on the rate of cell division most likely results from higher hexose levels, rather than from the amount of carbon import^6^. The filial tissue of a litchi seed accumulates about 90% dry mass during the filling stage (after 35 DAA), yet the silencing of *LcCWINs* at 35 DAA does not increase the seed abortion rate (Fig. 7). These results indicate that *LcCWIN5* functions in early litchi seed development when the endosperm and the embryo undergo active cell division.

In angiosperms, double fertilization leads to the formation of an embryo and an endosperm. The cellularized endosperm acts as nourishing tissue that is consumed by the embryo during maturation in dicotyledons^1^. If endosperm development fails, it will ultimately cause the embryo to arrest its development. Both endosperm and embryo are known to display high mitotic activity during early seed development^14^. We hypothesize that the impaired endosperm that resulted from decreased *CWIN* expression might be a consequence of nuclear division proceeding earlier in the endosperm than in the embryo (Figs. 1 and 7; Table 2). Our finding that the silencing of *LcCWIN5* at 28 DAA, when there is an abundance of liquid endosperm, resulted in failed embryo development (Fig. 8) indicates that the developing embryo can be directly controlled by CWIN.

Numerous studies have shown that sugar acts as a signaling molecule^27–29^. For example, in *A*. *thaliana*, sugar provides an endogenous cue for the juvenile-to-adult phase transition^30^. Sugar signals are perceived and transduced through a glucose sensor, HEXOKINASE1 (HXK1), which exerts its regulatory function through transcriptional activation, translational inhibition, mRNA decay, and protein degradation^27^. The CWIN-elevated transgenic tomato plants exhibited higher activities of hexokinase and fructokinase in ovaries and enhances fruit set through suppressing programmed cell death in the placenta under heat stress^31^. Litchi seed abortion under reduced CWIN might also be associated with the programmed cell death of the filial tissues. The underlying networks in early seed development of litchi in response to CWIN availability will provide a subject for future investigation.

### Different roles for the *LcCWIN* genes in seed development

In the present study, we identified and characterized five litchi *CWIN* genes. Except for LcCWIN1, the deduced amino sequences of the other four LcCWIN proteins all contain the highly conserved β-fructosidase motif and cysteine catalytic sites (Fig. 3). A phylogenetic tree of CWIN sequences from other plants and these four putative litchi CWIN proteins showed four clusters (monocot-I, monocot-II, dicot-I, and dicot-II), which appeared to be associated taxonomic relationship of the associated plants (Fig. 4). The LcCWIN2 gene belonged to dicot-I, while LcCWIN3-5 grouped with other dicot-II sequences, suggesting functional divergence. *LcCWIN2* was predominantly expressed in the funicle and seed coat, the site of seed assimilate unloading. *LcCWIN5* was specifically expressed in flowers (anther and pistil) and in young fruit (Figs. 5 and 6), which is consistent with of the expression pattern of *LIN5*, a cell wall invertase critical for seed development and fruit set in tomato^8^.

We adopted a VIGS approach to silence the expression of LcCWIN2/5 genes. As mentioned earlier, the silencing of *LcCWIN5* before 28 DAA significantly inhibited seed development and increased the rate of seed abortion of normal seed cultivars, whereas silencing of *LcCWIN5* after 28DAA did not affect seed development (Table 2, Figs. 7 and 8). These results suggest that *LcCWIN5* regulates litchi seed development at the early stage, when filial tissues undergo active cell division. The filial tissues (embryo and endosperm) are symplastically isolated from the maternal seed coat, and so sugars are secreted from the maternal layers and imported into the filial tissues. In maize seeds, an in situ fluorometric assay of glucose in tissue sections suggested that glucose might enter the filial tissues during the very early stages of seed development, 0–2 days after pollination^32^. LcCWIN5 was expressed in very young fruits (Fig. 6a). In litchi, CWIN activity in the critical sugar transportation site (funicle and seed coat) during the early stage of seed development is likely due to *LcCWIN5* expression, which helps the entrance of hexose, a prerequisite for normal seed development.

We observed high expression of *LcCWIN1*, which has deletions in an important functional motif, but low expression of *LcCWIN2* in tissues of the FZX cultivar (Fig. 6). The RT-PCR analysis showed that *LcCWIN2* transcript levels in the funicle of FZX were much lower than in NMC and HY at 28–42 DAA, when early seed filling occured (Figs. 1 and 6). This was consistent with the low CWIN activity in the funicle of FZX during this period (Fig. 2g). The CWIN activities in the seed coat and cotyledon of FZX during this period were also much lower than those in cultivar HY (Fig. 2g). In rice, GIF1 (GRAIN INCOMPLETE FILLING 1, also known as OsCIN2) is a cell wall invertase required for carbon partitioning during early grain-filling^7^. The *gif1* mutant is morphologically normal, with normal seed setting, but reduced grain weight and loosely packed starch granules^7^. Similarly, the *LcCWIN2* reduced cultivar FZX displayed normal liquid endosperm development, but reduced seed size (Figs. 1 and 6). And in addtition, the silencing of *LcCWIN2* reduced the seed size without increased abortion rate (Table 2, Fig. 8). Taken together, these results suggest that low expression of *LcCWIN2*, and thus low CWIN activity, during the critical seed filling stage might be associated with the small seed size of the FZX cultivar.

In the present study, the CWIN activities in the funicle, seed coat, and cotyledon of small-seeded FZX were all significantly lower than the big-seeded HY (Fig. 2g). In paralleled with the reduced seed size in response to the silencing of *LcCWIN2*, the CWIN activity in the cotyledon, but not in the funicle or seed coat, was significantly downregulated (Fig. S2). These results suggested that the CWIN in the filial tissue might play an important role in the seed development of litchi. This is consistent with the result of maize. A loss-of-function mutation at the Mn1 locus results in only 1% CWIN activity in the endosperm and a loss of >70% weight at maturity but non-lethal seed^6^.

In summary, we propose that the litchi CWIN genes have different roles in seed development. The maternal early-expressed *LcCWIN5* is involved in the early liquid endosperm and embryo development, whereas the late-expressed *LcCWIN2* is associated with seed filling.

Interestingly, the expression patterns of *LcCWIN5* among cultivars during the early stage of fruit development did not correlate with CWIN activity. Much lower CWIN activity, but comparable *LcCWIN5* expression, was detected in the funicle of the NMC cultivar (Figs. 2 and 6). This suggests that post-translational regulation may be responsible for the decreased CWIN activity and abnormal early seed development in NMC. This is in agreement with reports of post-translational regulation of invertase activity by inhibitory proteins in tobacco^33^, maize^34^, and tomato^12^. Future studies will focus on a cell wall invertase inhibitor gene (LcCIF, KX981211) that we have identified, which is predominantly expressed in NMC.

## Materials and methods

### Plant materials

The experiments were conducted at the experimental orchard of the South China Agricultural University, Guangzhou, China. The litchi trees used for the experiments received standard horticultural practices and were open pollinated. Mature leaves, male and female flowers, and fruits from three trees were collected from big-seeded cultivar “Heiye,” small-seeded cultivar “Feizixiao,” and seed-aborted cultivar “Nuomici.” The funicle, seed coat, and cotyledon tissues of different litchi cultivars were harvested to measure the CWIN activities and the expression of *LcCWIN* genes from anthesis until 49 DAA when cotyledons filled the seed coat.

For VIGS assay, the cultivars were chosen on the basis of their seed types. In 2015, big-seeded cultivars, “Heiye” and “9911,” was used to investigate the effects of *LcCWIN5* on seed development through fruit-stalk injection at 21 DAA. In 2016, the big-seeded cultivar, “Heiye,” and the partly aborting cultivar, “Baitangying,” were used to evaluate the effects of *LcCWIN2* and *LcCWIN5* silencing through panicle dipping at female blooming. Furthermore, the big-seeded cultivars, “Heiye” and “Shuilin,” were used to investigate the effects of *LcCWIN2* and *LcCWIN5* silencing on seed development through fruit-stalk injection at 21, 28, and 35 DAA.

### Observation of seed development and the measurement of liquid endosperm volume and seed weight

Fruit at different developmental stages were cut longitudinally into two parts and photographed. A syringe (100 μL) was used to remove and quantify the volume of liquid endosperm after cutting the tip of the seed. More than 50 fruits from each sampling tree were used for the liquid endosperm volume measurement. The seed weights of cultivars “Heiye,” “Feizixiao,” and “Nuomici” were measured at fruit maturity using 1/1000 electronic balance.

### CWIN activity assays and immunocolloidal gold labeling

Proteins were extracted and CWIN activity was assayed, as previously reported^35^. For the immunogold labeling assay, litchi funicles were cut into 2–3 mm^3^ cubes that were immediately fixed with 4% (w/v) paraformaldehyde and 2.5% (v/v) glutaraldehyde in 100 mM precooled PBS (8 mM Na_2_HPO_4_, 1.5 mM KH_2_PO_4_, 3 mM KCl, and 500 mM NaCl, pH 7.2) for 4 h. Dehydration and subsequent infiltration was conducted according to Zhang et al^36^. Ultrathin sections (~50 nm) were cut and mounted on 100-mesh nickel grids coated with 0.3% Formvar films for subsequent immunolabeling. The ultrathin sections were first blocked by floating the grids on droplets of PBS (pH 7.4) supplemented with 50 mM glycine for 30 min at room temperature and continuously blocked with PBS (pH 7.4) supplemented with 0.1% (w/v) gelatin, 0.5% (w/v) bovine serum albumin (BSA), and 0.1% (v/v) Tween 20. Antiserum against CWIN was custom made in Bioleaf Biotechnology Co., Ltd (Shanghai, China). Goat anti-rabbit IgG antibody conjugated with 10 nm gold was purchased from Sigma.

### Isolation and cloning of CWIN genes

Fourteen sequences annotated as putative invertase were obtained from the *L*. *chinensis* genome database (http://litchidb.genomics.cn/page/species/index.jsp). Among them, five sequences showed high similarity to the reported CWIN genes from other plant species and were predicted to encode proteins with conserved CWIN functional motifs. These were targeted as putative litchi CWIN genes and were named *LcCWIN1* to *LcCWIN5*. Their coding sequences were amplified from cDNA libraries using gene-specific primers (Table S1). Full-length *LcCWIN1*, *LcCWIN3*, and *LcCWIN4* fragments were obtained from a cDNA library that was reversed transcribed from litchi leaf mRNA, while full-length *LcCWIN2* and *LcCWIN5* were obtain from cDNA libraries that were reversed transcribed from funicle and flower mRNA extracts.

### Quantitative real-time PCR (qRT-PCR) analysis

Total RNA from different litchi organs/tissues was extracted using the RNA_OUT_ kit (Tiandz, Beijing, China), and cDNA was synthesized from total RNA (2 μg) using oligo dT primers and M-MLV reverse transcriptase, according to the manufacturer’s instructions (Invitrogen, USA), in a total volume of 20 μL. *LcCWIN* transcript levels were measured using qRT-PCR, as previously described^37^. The specific real-time PCR primers are listed in Table S2. We analyzed the expression in biological triplicate samples. Real-time PCR reactions were normalized to Ct values for litchi *LcActin* (HQ615689) and *LcGAPDH* (JF759907). The relative expression levels of the target genes were calculated using the 2^△△Ct^ method^38^.

### VIGS-mediated *LcCWIN* silencing

VIGS was carried out as previously described, with modifications in treatments and bacterial concentration^25^. Air temperature and humidity is critical for high infection rate, and therefore successful for VIGS assays. The orchard air temperature is typically 22–25 °C and the relative humidity is usually > 70% during litchi flowering and young fruit development, which is optimal for VIGS assays in the orchard. TRV1 and TRV2 vectors containing *LcCWIN2* (400 bp) or *LcCWIN5* (446 bp) fragments were transformed separately into *Agrobacterium tumefaciens* strain GV3101 and then grown separately overnight in LB liquid media containing 50 mg L^−^^1^ kanamycin and 1 mg L^−1^ rifampicin. The rest of the protocol followed our previous report^25^. After optimization, two ways of treatment, panicle dipping and fruit-stalk injection, were employed for silencing of the genes, with a bacterial culture concentration of OD_600_ ≈ 1.0. For the dipping treatments, panicles were dipped into the bacterial suspension for about 30 s at female flower blooming. For the injection treatments, a 1 mL syringe was used to inject bacterial suspension into the fruit stalk. Thirty inflorescence or clusters existing in different parts of the canopy of each tree were treated. Among them, 20 clusters were used to investigate the fruit retention, seed weight, and abortion rate, and the rest ten clusters were used to take samples to measure liquid endosperm volumes and silencing effects. The number of fruitlets was counted both at 2 weeks after panicle dipping or at the day of injection and 49–56 DAA, and the fruit retention rate was calculated. The liquid endosperm volume was investigated at around 28 DAA or 10 days after injection. The seed weight and abortion rate were investigated around 49–77 DAA, when seed filling had finished or at fruit maturity. Samples were collected ~5 weeks after dipping to test the silencing effects. Three fruits from three individual clusters were pooled into a single replication.

### Sequence and statistical analyses

Multiple sequence alignment was performed using ClustalX 1.83 (http://www.ebi.ac.uk) and MEGA5^39^. The signal peptide, putative cleavage site, and putative glycosylation sites were predicted using the Signal P 3.0 server, NetNGlyc 1.0 Server (http://www.cbs.dtu.dk/services/NetNGlyc/), and the DictyOGlyc server (http://www.cbs.dtu.dk/index/shtml-/), respectively.

Data were processed with SigmaPlot 10.0. Statistical analyses were performed using the statistical DPS (v3.0) package. A two-tailed *t*-test or Duncan multiple range test were used to determine significance at the 5% level.

## Electronic supplementary material

Table S1

Table S2

Figure S1

Figure S2

Supplementary data

## References

[1] Lafon-Placette C, Köhler C (2014). Embryo and endosperm, partners in seed development. Curr. Opin. Plant Biol..

[2] Wang L, Liao S, Ruan YL (2013). Cell wall invertase as a regulator in determining sequential development of endosperm and embryo through glucose signaling early in seed development. Plant Signal. Behav..

[3] Weber H, Borisjuk L, Wobus U (1996). Controlling seed development and seed size in *Vicia faba*: a role for seed coat-associated invertases and carbohydrate state. Plant J..

[4] Weber H, Borisjuk L, Wobus U (1997). Sugar import and metabolism during seed development. Trends Plant Sci..

[5] Weschke W (2003). The role of invertases and hexose transporters in controlling sugar ratios in maternal and filial tissues of barley caryopses during early development. Plant J..

[6] Chourey PS, Jain M, Li QB, Carlson SJ (2006). Genetic control of cell wall invertases in developing endosperm of maize. Planta.

[7] Wang ET (2008). Control of rice grain-filling and yield by a gene with a potential signature of domestication. Nat. Genet..

[8] Zanor MI (2009). RNA interference of LIN5 in tomato confirms its role in controlling Brix content, uncovers the influence of sugars on the levels of fruit hormones, and demonstrates the importance of sucrose cleavage for normal fruit development and fertility. Plant Physiol..

[9] Wang L, Ruan YL (2012). New insights into roles of cell wall invertase in early seed development revealed by comprehensive spatial and temporal expression patterns of *GhCWIN1* in cotton. Plant Physiol..

[10] Tang X (2017). Suppression of extracellular invertase inhibitor gene expression improves seed weight in soybean (*glycine max*). J. Exp. Bot..

[11] Miller ME, Chourey PS (1992). The maize invertase-deficient miniature1 seed mutation is associated with aberrant pedicel and endosperm development. Plant Cell.

[12] Jin Y, Ni DA, Ruan YL (2009). Posttranslational elevation of cell wall invertase activity by silencing its inhibitor in tomato delays leaf senescence and increases seed weight and fruit hexose level. Plant Cell.

[13] Fridman E, Carrari F, Liu YS, Fernie AR, Zamir D (2004). Zooming in on a quantitative trait for tomato yield using interspecific introgressions. Science.

[14] Weber H, Borisjuk L, Wobus U (2005). Molecular physiology of legume seed development. Annu. Rev. Plant Biol..

[15] Zhang Y, Zhang A, Jiang J (2013). Gene expression patterns of invertase gene families and modulation of the inhibitor gene in tomato sucrose metabolism. Genet. Mol. Res..

[16] Kim JY (2000). Characterization of two members of the maize gene family, *Incw3* and *Incw4*, encoding cell-wall invertases. Gene.

[17] Bocock P, Morse A, Dervinis C, Davis J (2008). Evolution and diversity of invertase genes in *Populus trichocarpa*. Planta.

[18] Cho JI (2005). Molecular cloning and expression analysis of the cell-wall invertase gene family in rice (*Oryza sativa* L). Plant Cell Rep..

[19] Roitsch T, González MC (2004). Function and regulation of plant invertases: sweet sensations. Trends Plant Sci..

[20] Yang ZY (2014). Does acid invertase regulate the seed development of *Litchi chinensis*?. Acta Hort..

[21] Lü LX, Chen JL, Chen XJ (1985). An observation on the process of embryo development in litchi. Subtrop. Plant Res. Comm..

[22] Wang TD (2015). Sugar uptake in the aril of litchi fruit depends on the apoplasmic post-phloem transport and the activity of proton pumps and the putative transporter LcSUT4. Plant Cell Physiol..

[23] Roitsch T, Bittner M, Godt DE (1995). Induction of apoplastic invertase of *Chenopodium rubrum* by D-glucose and a glucose analog and tissue-specific expression suggest a role in sink-source regulation. Plant Physiol..

[24] Roy KL (2013). Understanding the role of defective invertases in plants: tobacco nin88 fails to degrade sucrose. Plant Physiol..

[25] Li XJ (2016). Functional characterization of a glucosyltransferase gene, *LcUFGT1*, involved in the formation of cyanidin glucoside in the pericarp of *Litchi chinensis*. Physiol. Plant.

[26] Vilhar B, Kladnik A, Blejec A, Chourey PS, Dermastia M (2002). Cytometrical evidence that the loss of seed weight in the miniature1 seed mutant of maize is associated with reduced mitotic activity in the developing endosperm. Plant Physiol..

[27] Rolland F, Sheen J (2005). Sugar sensing and signalling networks in plants. Biochem. Soc..

[28] Smeekens S, Ma J, Hanson J, Rolland F (2010). Sugar signals and molecular networks controlling plant growth. Curr. Opin. Plant Biol..

[29] Lastdrager J, Hanson J, Smeeken S (2014). Sugar signals and the control of plant growth and development. J. Exp. Bot..

[30] Yu S (2013). Sugar is an endogenous cue for juvenile-to-adult phase transition in plants. eLife.

[31] Liu YH, Offler CE, Ruan YL (2016). Cell wall invertase promotes fruit set under heat stress by suppressing ROS-independant cell death. Plant Physiol..

[32] McLaughlin JE, Boyer JS (2004). Sugar-responsive gene expression, invertase activity, and senescence in aborting maize ovaries at low water potentials. Ann. Bot..

[33] Greiner S, Krausgrill S, Rausch T (1998). Cloning of a tobacco apoplasmic invertase inhibitor: proof of function of the recombinant protein and expression analysis during plant development. Plant Physiol..

[34] Bate NJ, Niu X, Wang Y, Reimann KS, Helentjaris TG (2004). An invertase inhibitor from maize localizes to the embryo surrounding region during early kernel development. Plant Physiol..

[35] Yang ZY (2013). Patterns of enzyme activities and gene expressions in sucrose metabolism in relation to sugar accumulation and composition in the aril of *Litchi chinensis* Sonn. J. Plant Physiol..

[36] Zhang LY (2004). Evidence for apoplasmic phloem unloading in developing apple fruit. Plant Physiol..

[37] Lai B (2014). LcMYB1 is a key determinant of differential anthocyan in accumulation among genotypes, tissues, developmental phases and ABA and light Stimuli in *Litchi chinensis*. PLOS One.

[38] Livak KJ, Schmittgen TD (2001). Analysis of relative gene expression data using real-time quantitative PCR and the 2(-Delta Delta C(T)) method. Methods.

[39] Tamura K (2011). MEGA5: Molecular evolutionary genetics analysis using maximum likelihood, evolutionary distance, and maximum parsimony methods. Mol. Biol. Evol..

