# Supplementary material for: Aberrant seed development in Litchi chinensis is associated with the impaired expression of cell wall invertase genes
Source: Hortic Res. 2018 Aug 1;5:39. doi: 10.1038/s41438-018-0042-1 (PMC6068106; doi:10.1038/s41438-018-0042-1)
Supplement: Supplementary file 1 — Table S1 [file 41438_2018_42_MOESM1_ESM.docx]

**Table S1.** Primers used for *CWIN* gene isolation.

| Gene | Forward primer  (5' to 3') | Reverse primer  (5'to 3') |
| --- | --- | --- |
| *LcCWINI* | ATGGCCTTCGTCATTAGAAACAG | CTATATCTTAAAACCTTCCTCTCCC |
| *LcCWIN2* | ATGGAGATATCAAAATATCTATCAG | CTAGTTCATCTCGACGGGC |
| *LcCWIN3* | ATGGCCAACCGCTCTACTTTC | TCAAAAAATATGGGCTTTCTTCATG |
| *LcCWIN4* | ATGACCAACTTCTCCGTTACTC | TCAGTTAATTTGAGCTTTCTTC |
| *LcCWIN5* | ATGGCTAACACTTCAATTTCTC | TCAATTGATTTGAGCTTTGTTC |
