# Supplementary material for: Aberrant seed development in Litchi chinensis is associated with the impaired expression of cell wall invertase genes
Source: Hortic Res. 2018 Aug 1;5:39. doi: 10.1038/s41438-018-0042-1 (PMC6068106; doi:10.1038/s41438-018-0042-1)
Supplement: Supplementary file 2 — Table S2 [file 41438_2018_42_MOESM2_ESM.docx]

**Table S2.** Primers used for real-time PCR.

| Gene | Forward primer  (5' to 3') | Reverse primer  (5'to 3') |
| --- | --- | --- |
| *LcCWINI* | GCAAGCAAGCCAGCATCAA | TCGGTCGCCAAAGCTAACA |
| *LcCWIN2* | GAACAAGGTCCTCAAGTCAG | CTAATGTCAAGAGCCCAAAT |
| *LcCWIN3* | TTCAGGCCGATGTGGAGAT | AAGCCAAAGCCAGCAACCC |
| *LcCWIN4* | CACTAAGGAGCTTGATTGACC | AGATTTGAGATGCTGACCC |
| *LcCWIN5* | TGGCTTCCTGACTTTCGC | TGCCGGGTCCACATCTAC |
