# Supplementary figures and images for: Aberrant seed development in Litchi chinensis is associated with the impaired expression of cell wall invertase genes
Source: Hortic Res. 2018 Aug 1;5:39. doi: 10.1038/s41438-018-0042-1 (PMC6068106; doi:10.1038/s41438-018-0042-1)

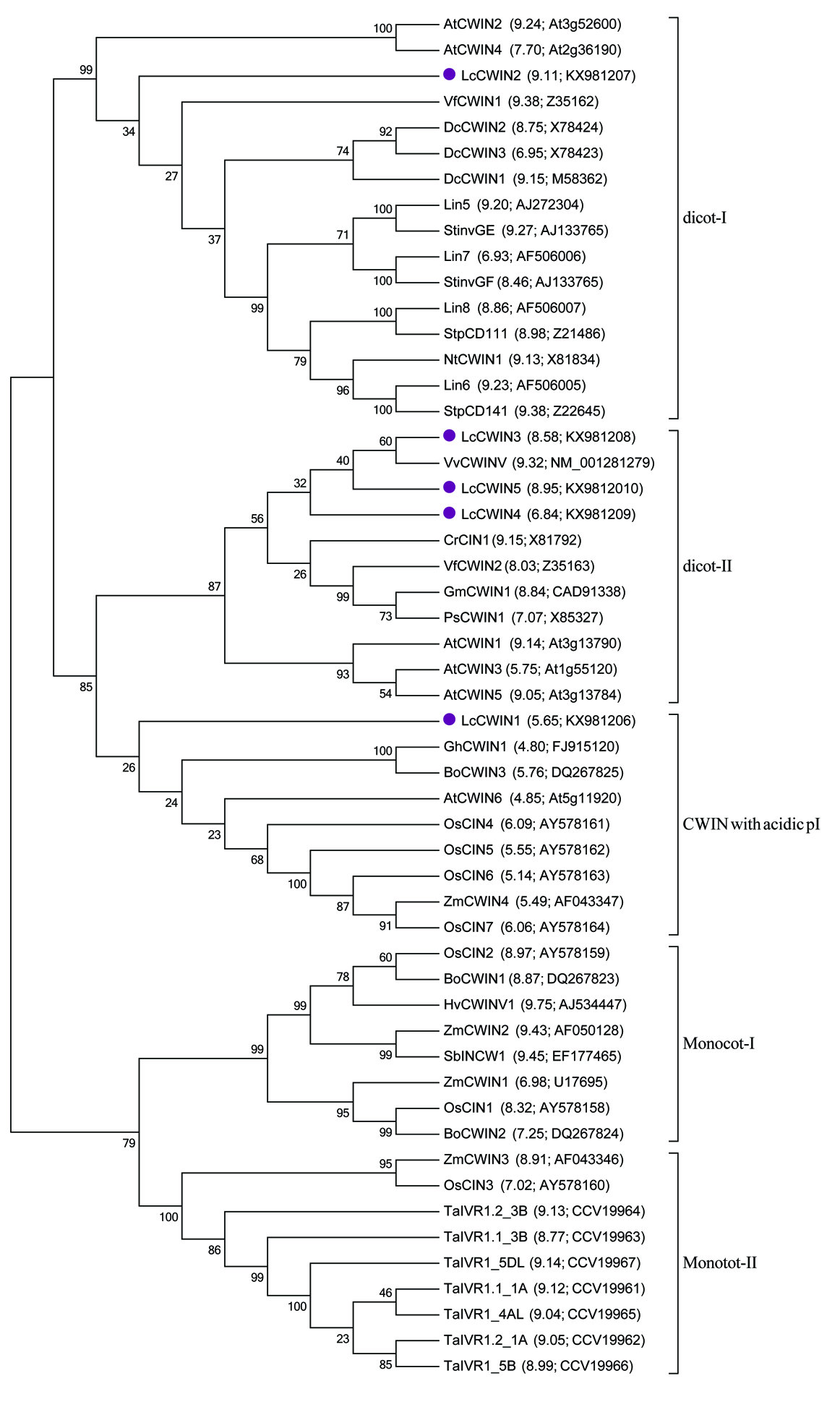

Supplement: Supplementary file 3 — Figure S1 [file 41438_2018_42_MOESM3_ESM.tif]

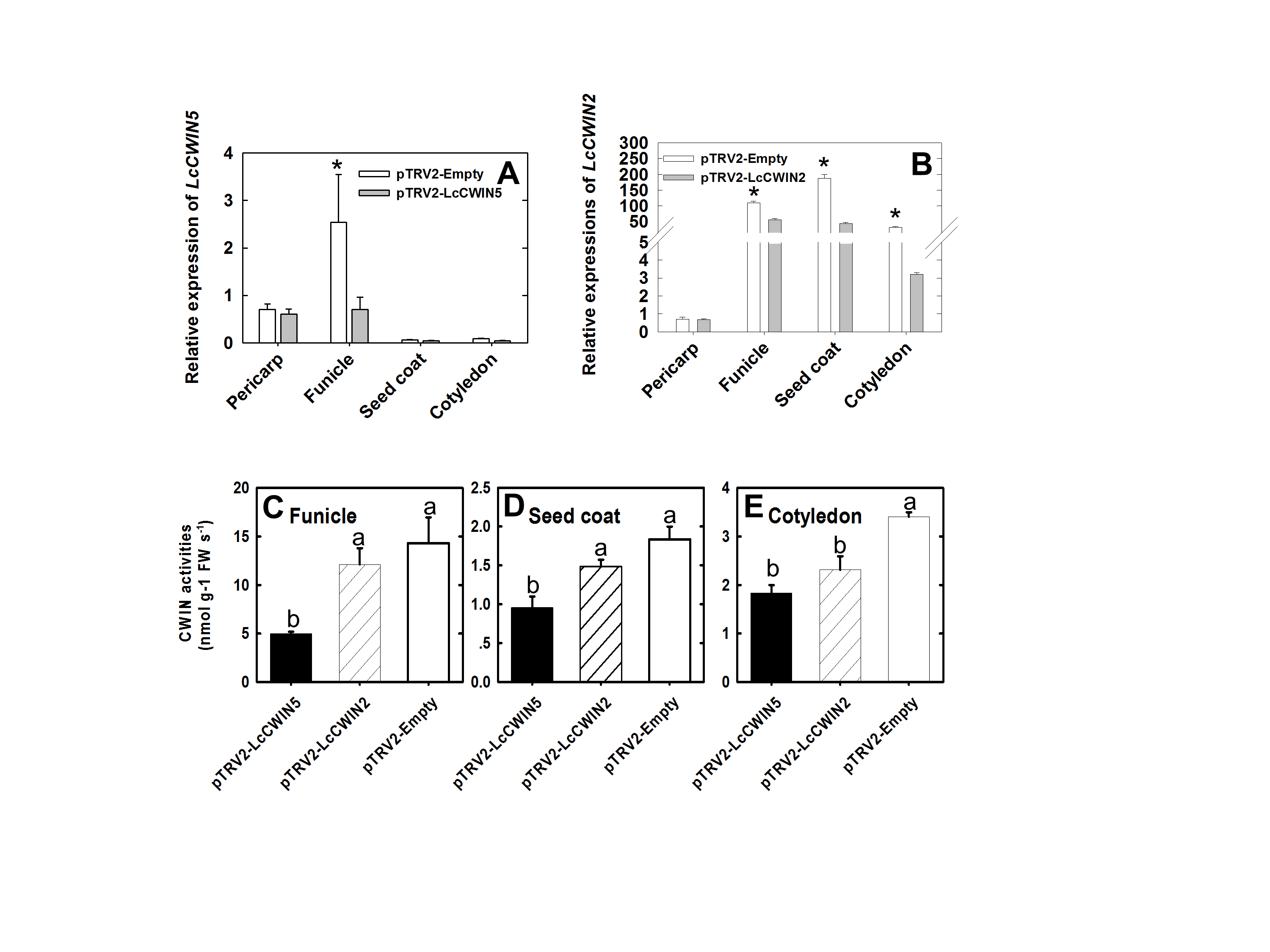

Supplement: Supplementary file 4 — Figure S2 [file 41438_2018_42_MOESM4_ESM.tif]
