# Supplementary material for: Aberrant seed development in Litchi chinensis is associated with the impaired expression of cell wall invertase genes
Source: Hortic Res. 2018 Aug 1;5:39. doi: 10.1038/s41438-018-0042-1 (PMC6068106; doi:10.1038/s41438-018-0042-1)
Supplement: Supplementary file 5 — Supplementary data [file 41438_2018_42_MOESM5_ESM.docx]

**Supplementary data**

Supplementary data are available online.

**Table S1.** Primers used for *CWIN* gene isolation.

**Table S2.** Primers used for real-time PCR.

**Fig. S1.** Phylogenetic relationship between the LcCWIN proteins and CWIN proteins from other plant species. The phylogenetic tree was constructed using the neighboring-joining method with MEGA 5 following multiple sequence alignment with ClustalW, with 1,000 bootstrap replicates. For each protein, the pI value and accession number are provided in parentheses. Species abbreviations are described in Fig. 4.

**Fig. S2.** Real-time PCR and CWIN activity measurements confirming the silencing of *LcCWIN5* and *LcCWIN2*. A and B are real-time PCR of *LcCWIN5* and *LcCWIN2* respectively; C, D and E are CWIN activities in the funciles, seed coat and cotyledon, respectively. Significantly decreases in *LcCWIN5* expression in the funicle, and in LcCWIN2 expression in all tissues tested, excluding the pericarp, was detected. As observed a significant decrease in CWIN activity in response to virus-induced *LcCWIN5* silencing. The CWIN activities in the funcile and seed coat were reduced but did not reach significant level, while CWIN activity in the cotyledon was significantly decreased in response to virus-induced LcCWIN2 silencing. * represents significance at p<0.05 using T-test (n=3). Different letters above the bars represent significant differences at P<0.05 (n=3, Duncan’s multiple range test).
